# Does digital inclusive finance promote the integration of rural industries? Based on the mediating role of financial availability and agricultural digitization

**DOI:** 10.1371/journal.pone.0291296

**Published:** 2023-10-10

**Authors:** Yan Huang, Junyan Zhao, Shi Yin

**Affiliations:** College of Economics and Management, Hebei Agricultural University, Baoding, 071001, China; Shenzhen University, CHINA

## Abstract

The integration of rural industries will inevitably lead to new business forms and new models, which put forward new requirements for traditional agricultural finance. The development of digital inclusive finance will provide new momentum for the integration of rural industries. Based on the provincial panel data from 2011 to 2020, the evaluation index system is constructed from three dimensions: industrial integration method, integration subject and integration format, and the development index of rural industrial integration is calculated. This paper establishes double fixed effect model and intermediary effect model to test the effect and path of digital inclusive finance on the integration of rural industries, and further explores the regulatory role and spatial difference of financial support. The results show that: (1) The integration of rural industries shows a growing trend, the eastern region develops more rapidly, while the central and western regions develop more slowly; (2) The digital inclusive finance can promote the integration of rural industries, digitization degree is remarkable, but coverage breadth and using depth are not significant, increasing the rate of per capita electricity consumption and urbanization can promote the integration of rural industries, consumption has limited pulling effect on the integration of rural industries, the per capita investment in fixed assets has no significant effects on the integration of rural industries; (3) The financial availability and the agricultural digitization play a complete intermediary effect; (4) Financial support has a negative moderating effect on the relationship between the two; (5) The eastern and central regions have a significant promoting effect, while the western region has a negative effect.

## 1. Introduction

The integration of rural industries refers to the extension of agriculture to secondary and tertiary industries, the cross-fertilization between industries, the formation of new dynamic energy, new business models and new modes [1], a more perfect interest linkage mechanism for each subject in the industrial chain, and a higher degree of integration between one, two and three industries, ultimately realizing farmers’ income and rural industrial revitalization. With the strong support of the state, the integration of rural industries is effective, new models and new business models are emerging, and the integration of the main body continues to grow. But now, the integration of rural industries is still in the primary stage, the extension of the industrial chain is not sufficient, and the bottleneck of capital elements is prominent [2]. The integration of rural industries requires large and long-term financial support, and it also requires integrated consideration and synergistic promotion of different demand level subjects such as new agricultural business subjects and traditional small farmers in the industry chain [3], which presents new challenges and opportunities for financial innovation. Compared with the new agricultural business entities, small farmers have a small scale of operation and it is more difficult to obtain financing. Klose and Outlaw (2005) suggested that in order to solve the diversified financing needs faced by the integration of rural industries, it is necessary to optimize the rural financial service system, broaden financing channels, and innovate financial products to provide financial support for industrial development [4]. Ngozi et al. (2020) argued that the access of farmers to financial support is conducive to the integration of rural industries and that priority should be given to the role of finance in supporting agriculture and thus inducing industrialization [5]. Kelani et al. (2020) found that the agricultural sector is underfunded and that traditional agricultural financing does not contribute much to the economy, hindering the development of agriculture [6]. Compared with traditional finance, digital inclusive finance relies on digital technology and has unique advantages such as low cost, low risk, wide coverage, efficiency and convenience [7]. It innovates financial services to support agriculture, uses big data to promote the construction of rural credit system, promotes the extension of industrial chain and improves the benefit linkage mechanism. So, does digital inclusive finance promote the integration of rural industries in practice? How to clarify the paths and constraints? At present, digital inclusive finance is in the stage of development and improvement, and the study of these questions can help policy makers scientifically utilize digital financial resources, improve the financial dilemma of rural industrial integration, promote the development of the whole agricultural industry chain, and accelerate the modernization of agriculture and rural areas.

With the continuous innovation and development of digital technology, the combination of fintech and inclusive finance to provide inclusive financial services to disadvantaged groups, digital inclusive finance has significant advantages and an important impact on all aspects of the social economy [8]. Klapper and Singer (2015) found that digital inclusive finance can effectively contribute to the growth of residents’ assets and thus economic growth with its unique convenience and accessibility [9], and Lagna and Ravishankar (2022) study found that fintech can promote inclusive economic growth [10]. Jin (2017) [11], Mushtaq and Bruneau (2019) [12] argued that digital inclusive finance can have a significant poverty reduction effect, and Lauer and Lyman (2015) pointed out that digital technologies can expand the effective reach of financial services, provide accessible and appropriate financial services and products to more low-income groups, and reduce the income gap [13]. In addition, Tang et al. (2019) found that digital inclusive finance can accelerate industrial structure upgrading [14], Zou and Huang (2021) argued that digital inclusive finance can enhance regional innovation capacity [15], and Zhang and Wang (2021) argued that digital inclusive finance plays an important role in promoting high-quality agricultural development [16].

Scholars have explored the impact path of digital inclusive finance on the integration of rural industries from different perspectives. Zhang and Wen (2022) found that improving payment convenience and alleviating liquidity constraints are the main paths of digital inclusive finance for the integration of rural industries [17]. Zhang and Zhou (2021) proposed that digital inclusive finance improves the lack of capital in rural areas by broadening financing channels, innovating financial services, and stimulating the reform of traditional financial services [18]. Gao and Teng (2021) proposed that digital inclusive finance uses Internet technology to improve total factor productivity, promote cross-sectoral distribution of factors, and enhance the rationalization of industrial structure [19]. At the level of empirical analysis, Cao and Zhang (2023) used a systematic GMM approach and a threshold effect model to verify that digital inclusive finance can significantly promote the integration of rural industries in China [20]. In terms of indicator selection, regarding the measurement of digital inclusive finance, Peking University Digital Inclusive Finance Index is more authoritative and widely used by scholars [21]. The rural industry integration index is expressed by a single indicator, such as Liu et al. (2021) selected the proportion of agriculture, forestry, animal husbandry and fishery services to represent the development status of rural industry integration [22]. Some also use evaluation models such as entropy value method, principal component analysis, and hierarchical analysis to measure the comprehensive index, such as Tan and Yao (2021) used the entropy value method to measure the comprehensive index from three dimensions: extension of agricultural industry chain, integrated development of agricultural service industry and multifunctionality of agriculture [23]. Research on the impact of digital inclusive finance on the integration of rural industries has been little studied but importantly explored by scholars at home and abroad. Possible academic marginal contributions of this paper: to include financial accessibility and agricultural digitization as mediating variables in the research framework of the relationship between the two, to analyze their effects on the integration of industry chain subjects and mechanisms from the perspective of risks and costs, and to explore the moderating role of financial support to agriculture on the relationship between the two, and to further clarify the path of the role of digital inclusive finance on the integration of rural industries.

The research of this paper is as follows: Section 2 analyses the mechanism of the impact of digital inclusive finance on the integration of rural industries based on theoretical foundations and proposes research hypotheses. Section 3 is the methodology, including model construction, variable selection and data sources. Section 4 is an empirical study, constructing a rural industry integration index, regressing digital inclusive finance on rural industry integration, further analysing whether the mediating effect of financial accessibility and agricultural digitization is significant, whether financial support plays a moderating role, and examining regional differences in regression by region. Section 5 provides conclusions and implications, presenting the deficiencies of the paper and future prospects.

## 2. Research hypothesis

### 2.1. The impact of digital inclusive finance on the integration of rural industries

Digital inclusive finance relies on digital technology to reduce the cost and risk of financial services, which can both enhance the willingness of financial institutions to supply and meet the financing needs of the majority of farmers [24], providing new solutions to crack the industrial financing problem.

Digital inclusive finance promotes the integration of industries by improving financial accessibility. High natural risks in agriculture, imperfect credit system in rural areas and lack of financial knowledge of rural residents have led to serious financial exclusion in rural areas [25], and lack of financial support for industrial development. Small farmers have insufficient access to credit, which restricts production and management [26]. New agricultural operators need diversified financing to develop agricultural processing industry and service industry, but traditional financial supply is insufficient [27], and the bottleneck of industrial integration capital elements is prominent. Digital inclusive finance pursues the goal of "inclusiveness", provides inclusive financial services to disadvantaged groups, and solves the problem of "difficulty in financing" caused by the lack of collateral, which can effectively alleviate financial exclusion in rural areas and enhance financial accessibility [28]. Therefore, it is easier for digital inclusive finance to provide financial support for the extension of industrial chains and cultivation of new business models, to strengthen new agricultural business entities [15], and to rely on the qualifications and credit of core business entities in the agricultural industry chain, to link transactions between various entities in the upper and lower reaches for overall crediting of the industry chain, to promote the coordinated operation of the industry chain as a whole, to prevent market risks and strictly control the credit risks of farmers, and to improve the mechanism of linking interests [29]. The efficient use of financial resources can also enhance the level and allocation efficiency of factor inputs such as technology, manpower and land, providing factor security for the integration of rural industries, and agricultural finance can enhance technology adoption by farmers [30], digital inclusive finance can enhance human capital [31], accelerating rural land transfer [32], improving infrastructure construction and promoting efficient operation of industrial integration.

**Fig 1:**
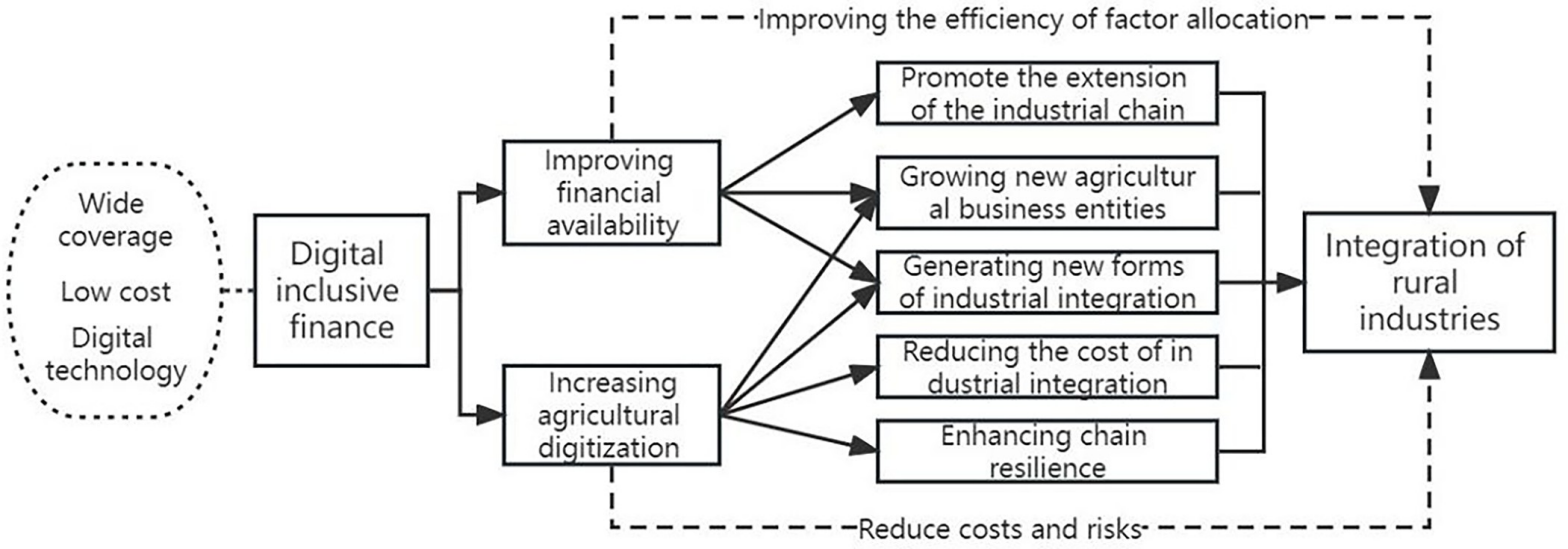
The influence mechanism of digital inclusive finance on the integration of rural industries.

Digital inclusive finance promotes industrial integration by enhancing the digitization of agriculture. The core of digital inclusive finance is data [33], by integrating the information data of agricultural business entities and establishing a database to provide information data for farmers’ production and operation and help them make production decisions. Digital inclusive finance uses digital technology to innovate financial products, serve the main body of industrial integration, and enhance the digital management of agriculture [34], thus reducing the cost and risk of industrial integration. On the one hand, the wide application of digital technology can improve the transparency of information platforms and the efficiency of information use, which is conducive to reducing the transaction costs of agricultural production and marketing links, adjusting the boundaries between the internal operation of enterprises and market transactions, and expanding the scale of market transactions of agricultural intermediate input factors, thus promoting the integration of rural industries [35]. On the other hand, digital technology has improved the ability of agricultural operators to obtain information on production technology and market changes, improved the risk resistance of agricultural operators, and enhanced the resilience of industrial chains [36]. In addition, digital technology has given birth to new industries and new modes of industrial integration. Relying on Internet platforms and exploring new modes of online business, new industries such as live e-commerce are developing rapidly [37], the new scenario of digital consumption is widely applied. The mechanism of digital inclusive finance affecting the integration of rural industries is shown in Fig 1. The following hypotheses are proposed in this paper, hypothesis 2 and hypothesis 3 are based on hypothesis 1.

**Hypothesis 1.** *Digital inclusive finance can promote the integration of rural industries*.
**Hypothesis 2.** *Digital inclusive finance promotes the integration of rural industries by enhancing financial accessibility*.
**Hypothesis 3.** *Digital inclusive finance promotes the integration of rural industries by enhancing agricultural digitization*.

### 2.2. The moderating effect of financial support on the relationship between digital inclusive finance and the integration of rural industries

The development of rural industrial integration has strong positive externalities and is an important area for financial support [38]. Therefore, an in-depth discussion on the heterogeneous impact of financial support on the relationship between the two is an important direction to grasp the path of action. The integration of rural industries requires a large amount of financial support, and the guidance and leverage of financial funds can guide and leverage the flow of social funds to projects needed for the development of rural industries [39]. Huang (2021) found that agricultural fiscal policies can optimize the allocation of resources, promote the flow of factors and support the development of agricultural industrialization [40]. Hu and Ding (2019) argued that fiscal subsidies can bring value-added to the industrial chain [41]. The construction of industrial integration parks and the extension and expansion of industrial chains require large amounts of capital investment, and farmers’ own funds are limited, so financial coordination and cooperation are needed to jointly support the deep integration of agriculture and secondary and tertiary industries. Financial support for the integration of rural industries is inseparable from the coordination and cooperation of finance. Continued financial investment, playing a guiding and encouraging role, will prompt the flow of financial resources to the needs of industrial development and provide a constant stream of support for industrial integration. By playing a good regulatory role, finance can highlight the advantages of digital inclusive finance to help agriculture, make up for the shortcomings of traditional finance and provide new solutions to address the new needs arising from industrial financing.

However, due to the long implementation and operation cycle of rural industrial integration projects at this stage, the effect of financial subsidies to boost digital financial support for industrial integration is difficult to be seen in the short term. In addition, the problem of rent-seeking in rural areas in China is serious, and the low level of financial support and imperfect subsidy mechanism have reduced the efficiency of financial fund use [42], the guiding effect on industrial development is limited. Gong and Gong (2021) found that there were many problems with financial support for agricultural industrialization, which not only did not promote the development of agriculturalization but also hindered the growth of the rural economy [43]. When financial support and finance are not well coordinated, the extension and expansion of industrial chains are not supported by funds, and agricultural operators are not motivated to produce, the development of rural industrial integration will be hindered. The result of financial support on the relationship between the two depends on the contrast of different forces: firstly, the inducement mechanism of financial input is conducive to the development of digital inclusive finance and promotes the deepening of industrial integration. Secondly, the imperfect mechanism of financial subsidy hinders digital inclusive finance from promoting the integration of rural industries. Therefore, there is uncertainty in the regulation of the relationship between digital inclusive finance and the integration of rural industries by financial support. Therefore, this paper puts forward the following hypothesis:

**Hypothesis 4.** *Financial support affects the effect of digital inclusive finance on the integration of rural industries*.

### 2.3. Spatial variability analysis

China is a vast country with different agricultural production conditions and rural economic development levels in different regions, and the degree of digital inclusive finance application and the integration of rural industries also show regional differences [44]. In general, the eastern region is experiencing rapid economic development, continued strong financial and fiscal investment, high levels of agricultural modernization and a leading level of rural industrial integration. At the same time, the financial industry in the eastern region is developed, digital technology is widely used, digital inclusive finance is more well developed, and rich financial resources provide strong support for industrial development [45]. Compared with the eastern regions, the central and western regions have fragile ecological environments, a lack of natural resources, slower agricultural development, backward development of rural industries and various realistic obstacles to industrial integration. In addition, the infrastructure facilities in the central and western rural areas are not yet complete, financial inhibitions are highlighted and digital inclusive finance is not widely used. In particular, the western region has a late start in development, with slow economic development, a weak agricultural base and a lack of industrial resources. Farmers have a low level of education, weak financial knowledge and credit awareness, insufficient understanding of financial products, weak credit awareness, inability to make reasonable use of financial tools and distrust of digital products, which in turn exacerbates the digital divide.

Ge and Li (2022) compared the differences in the impact of digital inclusive finance on rural industrial integration in different provinces and found the most significant impact in the eastern region [46]. In view of the development differences between digital inclusive finance and rural industrial integration, the agricultural foundation and economic development level of each province are different, and the influence of digital inclusive finance on rural industrial integration should be discussed in different regions [47]. The eastern digital inclusive finance has developed rapidly, and technologies such as big data and blockchain are widely used, farmers are relatively well educated, have certain financial literacy, and have strong ability to use digital equipment. when making production and operation decisions, they can rationally use digital financial products, expand their industries, and give full play to the advantages of digital inclusive finance in supporting agricultural products processing and circulation industry and cultivating new formats. Central and western China, due to the scarcity of financial resources, the small variety of financial products and services and the high threshold, the weak credit awareness of farmers, who are less likely to use the financing platform for production inputs and only use their own funds for production and operation, slow down the process of agricultural industrialization and have a limited role in promoting the integration of rural industries. Therefore, this paper puts forward the following hypothesis:

**Hypothesis 5.** *Spatial variability in the effects of digital inclusive finance on the integration of rural industries*.

## 3. Methodology

### 3.1. Model construction

Double fixation effect includes both time fixation effect and individual fixation effect, which means that it does not change with time or individual. To test whether digital inclusive finance has an impact on the development of rural industrial integration, a double fixed effects model is constructed as:

$$RI_{it}=\alpha_{0}+\alpha_{1}DIF_{it}+\alpha_{2}X_{it}+\upsilon_{i}+\theta_{t}+\varepsilon_{it}$$ (1)

To test the mediating effect of financial accessibility, Eqs (2) and (3) are added to Eq (1):

$$M_{it}=b_{0}+\beta_{1}DIF_{it}+\beta_{2}X_{it}+\nu_{i}+\theta_{t}+\varepsilon_{it}$$ (2)

$$RI_{it}=c_{0}+\gamma_{1}DIF_{it}+\gamma_{2}M_{it}+\gamma_{3}X_{it}+\upsilon_{i}+\theta_{t}+\varepsilon_{it}$$ (3)

To test the moderating effect of financial support, the model was constructed as:

$$RI_{it}=d_{0}+\delta_{1}DIF_{it}+\delta_{2}X_{it}+\delta_{3}DIF_{it}\times GOV_{it}+\nu_{i}+\theta_{t}+\varepsilon_{it}$$ (4)

In Eqs (1)–(4), *i* denotes province, *t* denotes time, RI denotes rural industrial integration and DIF denotes digital inclusive finance. The mediating variable is *M*_*it*_, which includes the financial accessibility of farm households (FA) and agricultural digitization (AD). The moderating variable is financial support to agriculture (GOV). *X*_*it*_ is a series of control variables, including electricity consumption per capital (Electricity), urbanization rate (UR), fixed asset investment per capital (FLX), and consumption level of rural residents (Consume), *ν*_*i*_ as individual fixed effects, *θ*_*t*_ as time fixed effects, and *ε*_*it*_ as random disturbance terms.

### 3.2. Variable selection and data sources

#### 3.2.1. Explained variables

The index of integration of rural industries (RI) is the explanatory variable. The index is constructed from the three dimensions of industry integration method, integration subject and integration mode, and the indicators are set as shown in Table 1. The first is integration method, including the extension of the agricultural processing industry and the integration of agriculture and services. The extension of agricultural processing industry refers to the vertical extension of agriculture, creating a tightly connected agricultural industry chain, processing of primary agricultural products is the most basic way, therefore the length of the extension of the agricultural processing industry is reflected by dividing the "business income of agricultural and sideline food processing industry" by the "value added of the primary industry", measuring the degree of integration of agriculture with the tertiary sector by the share of "value added of agriculture, forestry, animal husbandry, fishing and services" in "value added of the primary industry. The second is integration subject. As farmers’ professional cooperatives play an active role in promoting the integrated development of rural industries, the number of professional farmers’ cooperatives per 10,000 people is used to indicate the degree of perfection of the benefit linkage mechanism. The third is integration mode. Facility agriculture is a new trend in the development of rural industries, and the ratio of "area of facility agriculture" to "area of cultivated land" is used to reflect the cultivation of new agricultural businesses. The data were obtained from China Rural Statistical Yearbook, China Agricultural Management Business Statistics Annual Report, China Agricultural Yearbook, China Industrial Statistical Yearbook and national greenhouse system data. 30 provinces, excluding Tibet, from 2011 to 2020 were used as the explanatory variables in the model for the rural industrial integration development index, and logarithms were taken in the empirical analysis to make the data distribution more even.

**Table 1:** Integration subject and integration mode, and the indicators.

	Dimensions	Indicators	Indicator Calculation	Direction
Index of integrated rural industrial development	Integration method	Extension of agricultural processing industry	Business income of agricultural and sideline food processing industry / value added of the primary industry	+
		Integration of agriculture and services	Value added of agriculture, forestry, animal husbandry, fishing and services / value added of the primary industry	+
	Integration subject	Improvement of benefit linkage mechanism	Number of professional farmers’ cooperatives / Number of village population	+
	Integration model	Cultivation of new agricultural businesses	Area of facility agriculture / area of cultivated land	+

The entropy method was used to measure the composite index, with the following steps:

① Standardization of data:

$$x{'}_{ij}=\frac{x_{ij}-\min(x_{j})}{\max(x_{j})-\min(x_{j})}\;(positive\;indicators)$$ (5)

② Calculate P, the share of indicator *j* in year *i*:

$$P_{ij}=\frac{x{'}_{ij}}{\sum_{\alpha }\sum_{i}x{'}_{ij}}$$ (6)

③ Calculate the information entropy of the *j* indicator:

$$e_{j}=-k\sum_{i}P_{ij}\ln(P_{ij}),k=\frac{1}{\ln(mn)},k>0,e_{j}\geq 0$$ (7)

④ Calculate the redundancy of the information entropy of the *j* indicator:

$$d_{j}=1-e_{j}$$ (8)

⑤ Calculate the weight of the *j* indicator:

$$\omega_{j}=\frac{d_{j}}{\sum_{j}d_{j}}$$ (9)

⑥ Calculate the overall score:

$$X_{ij}=x{'}_{ij}\times \omega_{j}$$ (10)

#### 3.2.2. Explanatory variables

The Peking University Digital Inclusive Finance Index [21] is used as the core explanatory variable and is the most widely used and mature set of indices in this research area. In this paper, we use the Digital Inclusive Finance Index for 30 provinces excluding Tibet from 2011–2020 as the core explanatory variables in the model, and divide it by 100 to do a regression in the empirical analysis.

#### 3.2.3. Intermediate variables

In order to explore whether digital inclusive finance can promote the integration of rural industries by improving financial accessibility and agricultural digitization, we introduce intermediary variables. The intermediate variables are financial availability and agricultural digitization: (1) Financial accessibility of farm households (FA), Expressed using the average amount of loans actually obtained by farm households (the amount of farm loans divided by the number of people in the village). Data from China Financial Yearbook, China Statistical Yearbook, China Rural Financial Services Report. (2) Agriculture digitization (AD). Considering that the level of digitization will ultimately be reflected through digital applications, the number of rural broadband access subscribers divided by the number of rural population is chosen to measure the level of agricultural digitization.

#### 3.2.4. Adjustment variables

To examine whether the impact of digital inclusive finance on the integration of rural industries is moderated by other factors, this paper introduces financial support to agriculture (GOV) as a moderating variable in the research framework, and tests whether financial support has a moderating effect on the impact of digital inclusive finance on the integration of rural industries in Eq (4). The data are obtained from the China Rural Statistical Yearbook, and are regressed after taking logarithms in order to make the data distribution flatter.

#### 3.2.5. Control variables

In this paper, the following control variables that may affect the integration of rural industries are selected: (1) Rural infrastructure development, including transport, electricity and water infrastructure, using electricity per capital as a proxy for electricity infrastructure given the availability of data [48]. (2) Urbanization rate (UR), the proportion of urban population to the total population at the end of the year, is used to reflect the urbanization process. (3) Fixed asset investment per capital (FLX), expressed as the amount of fixed asset investment divided by the number of rural people. (4) Consumption expenditure of rural residents (Consume), taking into account the pulling effect of consumption on the integration of rural industries, the consumption expenditure of rural residents is chosen as the control variable. The data on per capital electricity consumption, urbanization rate, per capital fixed asset investment and rural residents’ living consumption expenditure were obtained from China Statistical Yearbook and China Rural Statistical Yearbook.

## 4. Empirical study

Firstly, the rural industrial integration index is constructed as the explained variable. Then, the explained variables and explanatory variables are used for baseline regression. On the basis of baseline regression, the intermediary variables are added for mediating effects, and the adjustment variable is added for mediating effects. The eastern, central and western regions are respectively subjected to baseline regression to test regional differences.

### 4.1. Rural industry integration index construction

The results of the rural industrial integration development index measurement are shown in Table 2. Overall, the integrated development of rural industries shows a growth trend, with the average value of the integrated index increasing from 0.0932 in 2011 to 0.1599 in 2020, a growth rate of 71.57%. There are significant differences in the development speed of each province, with the fastest growth between 2011 and 2020 being in Jiangsu province, which increased from 0.0906 in 2011 to 0.6591 in 2020, an increase of more than six times. There are also some provinces showing negative growth, such as Tianjin and Shandong, showing strong momentum at the beginning and insufficient momentum at the end. 2020, the development level of rural industrial integration is higher in the eastern provinces such as Jiangsu, Hainan and Shanghai, which have developed economies and strong capacity for agricultural science and technology innovation, and industrial integration has economic foundation and technical advantages, and industrial integration is at a higher level. Guangxi, Jiangxi, Heilongjiang, Jilin and other provinces are developing more slowly. Guangxi and Jiangxi have weak farmland foundation and low level of agricultural industrialization, while Heilongjiang and Jilin have superior agricultural production conditions, but low agricultural added value, insufficient extension of industrial chain and low level of industrial integration, and still have more room for development.

**Table 2:** Results of the rural industrial integration development index measurement.

Region	Province	2011	2012	2013	2014	2015	2016	2017	2018	2019	2020
Eastern regions	Beijing	0.1590	0.1600	0.1748	0.1703	0.1743	0.1929	0.1810	0.1767	0.1818	0.2103
	Tianjin	0.1865	0.2379	0.2395	0.2302	0.2725	0.2512	0.1986	0.1788	0.1587	0.1604
	Hebei	0.0864	0.0868	0.0885	0.0698	0.0928	0.0962	0.0986	0.1014	0.1065	0.1053
	Liaoning	0.1202	0.1334	0.1726	0.1606	0.1392	0.1195	0.1072	0.4009	0.3995	0.1852
	Shanghai	0.1299	0.1378	0.1505	0.1476	0.1422	0.1581	0.1655	0.3668	0.3579	0.3799
	Jiangsu	0.0906	0.0996	0.1087	0.1272	0.1331	0.1388	0.1399	0.6523	0.6561	0.6591
	Zhejiang	0.0694	0.0675	0.0697	0.0705	0.0735	0.0744	0.0770	0.2483	0.2534	0.2562
	Fujian	0.0679	0.0700	0.0726	0.0753	0.0786	0.0790	0.0820	0.0812	0.0803	0.0769
	Shandong	0.1223	0.1324	0.1380	0.1386	0.1392	0.1484	0.1308	0.1247	0.0925	0.0950
	Guangdong	0.0602	0.0615	0.0629	0.0636	0.0642	0.0647	0.0654	0.0656	0.0651	0.0661
	Hainan	0.1608	0.1550	0.1249	0.1412	0.1554	0.1674	0.1529	0.1490	0.1530	0.4519
Central regions	Shanxi	0.0864	0.0887	0.0876	0.0905	0.0952	0.0966	0.1024	0.1083	0.1153	0.1372
	Jilin	0.0899	0.1003	0.1058	0.1063	0.1065	0.1170	0.0915	0.0773	0.0704	0.0689
	Heilongjiang	0.0580	0.0579	0.0592	0.0585	0.0598	0.0614	0.0588	0.0610	0.0652	0.0647
	Anhui	0.0666	0.0689	0.0711	0.0739	0.0779	0.0833	0.0804	0.0811	0.0807	0.0818
	Jiangxi	0.0645	0.0634	0.0648	0.0674	0.0690	0.0691	0.0671	0.0676	0.0666	0.0664
	Henan	0.0656	0.0673	0.0711	0.0753	0.0829	0.0895	0.0817	0.0789	0.0788	0.0779
	Hubei	0.0593	0.0636	0.0753	0.0842	0.0873	0.0885	0.0888	0.0909	0.0895	0.0887
	Hunan	0.0660	0.0678	0.0710	0.0734	0.0761	0.0786	0.0802	0.0818	0.0779	0.0756
Western regions	Inner Mongolia	0.0485	0.0486	0.0599	0.0596	0.0600	0.0615	0.0606	0.0671	0.0918	0.0944
	Guangxi	0.0557	0.0563	0.0573	0.0584	0.0590	0.0596	0.0614	0.0632	0.0656	0.0671
	Chongqing	0.0544	0.0537	0.0645	0.0655	0.0671	0.0643	0.0651	0.0661	0.0680	0.0712
	Sichuan	0.0558	0.0580	0.0649	0.0640	0.0662	0.0653	0.0664	0.0694	0.0721	0.0737
	Guizhou	0.1415	0.1393	0.1131	0.1165	0.1301	0.1275	0.1578	0.1823	0.2138	0.2122
	Yunnan	0.1029	0.0956	0.0906	0.0882	0.0845	0.0773	0.0767	0.0783	0.0855	0.0952
	Shaanxi	0.0816	0.0808	0.0833	0.0833	0.0841	0.0848	0.0890	0.0905	0.0915	0.0941
	Gansu	0.0989	0.0948	0.0931	0.0951	0.0991	0.0890	0.1018	0.1118	0.1311	0.1269
	Qinghai	0.1442	0.1046	0.1147	0.0894	0.0801	0.0799	0.1028	0.1322	0.1810	0.2059
	Ningxia	0.1012	0.0959	0.1038	0.1039	0.1052	0.1074	0.1095	0.3449	0.3527	0.3370
	Xinjiang	0.1025	0.1007	0.0963	0.0946	0.0916	0.0924	0.1006	0.1096	0.1177	0.1119

### 4.2. Variable descriptive statistics

Descriptive statistics of the variables are shown in Table 3. The mean value of the rural industrial integration index is 0.1154, the maximum value is 0.6591 and the minimum value is 0.0485, with a large difference between the maximum and minimum values, indicating that the current development of rural industrial integration is uneven. The mean value of the Digital Inclusive Finance Index is 216.2352, while the maximum and minimum values are 431.9276 and 16.2200 respectively, indicating that the level of digital inclusive finance development varies greatly between provinces, and there is greater room for improvement in the development level of lagging regions. The level of financial accessibility and agricultural digitization are widely discrete. There are huge regional differences in financial support for agriculture and government support tilted towards remote areas. Per capital electricity consumption varies widely across provinces, with the maximum urbanization rate at 89.6% and the minimum at 35.03%. The maximum value of fixed asset investment per capital is 0.4841 and the minimum value is 0.0084, while the maximum value of consumption level of rural residents is 22,449 and the minimum value is 3,857.

**Table 3:** Descriptive statistics of variables.

Variables	Mean	Std. Dev.	Max	Min
RI	0.1154	0.0839	0.6591	0.0485
DIF	216.2352	96.8737	431.9276	16.2200
FA	1.1820	1.1446	10.3161	0.0752
AD	0.1319	0.1165	0.6292	0.0019
GOV	546.8481	269.9232	1339.3600	91.7800
Electricity	0.2367	0.6028	4.0868	0.0137
UR	59.0064	12.1979	89.6000	35.0300
FLX	0.1688	0.0700	0.4841	0.0084
Consume	10236.5967	3787.4653	22449.0000	3857.0000

### 4.3. Return to baseline

Before regression with a static panel model, the specific form of the model is tested, mainly the LR and Hausman tests. The LR test is carried out under a double fixed effects model and the p-value is less than the significance level, indicating that there is no mixed effect and a variable intercept model should be built. Then determine whether it is individual variation or time point variation, build a random effects model, carry out Hausman test, the p-value is less than the significance level, and finally build a double fixed effects model.

The baseline regression results are shown in Table 4, Model 1 shows the regression results of digital inclusive finance on the integration of rural industries, and Table 4 Model 2 adds the control variables of per capital electricity consumption, urbanization rate, per capital fixed asset investment and rural residents’ living consumption expenditure to the regression again, and the coefficients of digital inclusive finance (DIF) all pass the significance test, at 0.6034 and 0.4971 respectively, indicating that digital inclusive finance can promote the integration of rural industries and validate the previous hypothesis1. The coefficients of the other control variables were further analyzed: the coefficients of per capital electricity consumption and urbanization rate were positive and significant, indicating that the improvement of infrastructure and the acceleration of the urbanization process can promote the integration of rural industries. The coefficient of residents’ consumption level was negative, indicating that the pulling effect of consumption on industrial integration was limited and did not produce the expected effect. The coefficient of per capital fixed asset investment is not significant, probably due to the unreasonable structure of current fixed asset investment.

**Table 4:** Baseline regression.

Variables	Model 1	Model 2	Model 3	Model 4	Model 5
Constant	-3.6208*** (0.4428)	0.9345 (2.9901)	1.7789 (0.5988)	2.3457 (0.7762)	1.7138 (0.5862)
Electricity		0.2698*** (0.1005)	0.3012*** (3.0356)	0.3510*** (3.4469)	0.2751*** (2.8264)
UR		0.0212* (0.0117)	0.0090 (0.7411)	0.0118 (0.9448)	0.0264** (2.2446)
FLX		0.5362 (0.5095)	0.5797 (1.1321)	0.6874 (1.343086)	0.4889 (0.9694)
Consume		-1.4386* (0.7795)	-1.4567* (-1.8545)	-1.3451* (-1.7114)	-1.6368** (-2.1092)
DIF	0.6034*** (0.2024)	0.4971* (0.2211)			
Coverage breadth			0.5164 (1.5256)		
Using depth				-0.0894 (-0.6285)	
Digitization degree					0.2742*** (3.1991)
Time fixed	Yes	Yes	Yes	Yes	Yes
Individual fixed	Yes	Yes	Yes	Yes	Yes
Observed value	300	300	300	300	300
R^2^	0.7660	0.5739	0.7733	0.7716	0.7800

Note

***, **, * denote significant at the 1%, 5% and 10% levels respectively, standard deviations in brackets, below.

Model 3, Model 4 and Model 5 in Table 4 examine the effects of coverage breadth, using depth and digitization degree. It can be seen that the effect of digitization is significant, but the coverage breadth and using depth are not significant. Due to the rapid development of digital technology in the early stage, the digital advantage of digital inclusive finance is more significant [49], by providing convenient and low-cost financial services and thus promoting the integration of rural industries [50], but the coverage breadth and using depth are not enough, and the effect on the integration of rural industries is not significant.

### 4.4. Analysis of intermediary effects

The results of the regression of digital inclusive finance on the financial accessibility of farmers are shown in Table 5 Model 1, where the coefficient of digital inclusive finance is positively significant. Add in the digital inclusive finance and rural household financial availability to make a return to the integration of rural industries, the results are shown in Table 5 Model 2, the coefficient of digital financial inclusion is insignificant and the coefficient of financial accessibility is significant, indicating a full mediation effect, which verifies the previous hypothesis 2. With the development of digital inclusive finance, credit constraints in rural areas are gradually alleviated, more farmers receive loans, enhancing the accessibility of finance [51–53], meeting the financing needs of all subjects in the industry chain and further promoting the breadth and depth of rural industrial integration.

**Table 5:** Regression analysis of mediating effects.

Variables	Model 1 FA	Model 2 RI	Model 3 AD	Model 4 RI
Constant	11.5202* (2.0017)	-1.2203 (-0.4332)	-0.4278 (-0.8270)	1.3828 (0.4687)
Electricity	0.0024 (0.0124)	0.2694*** (2.8667)	-0.0024 (-0.1384)	0.2724 (2.7495)
UR	0.1105*** (4.9274)	0.0005 (0.0459)	0.0156*** (7.7620)	0.0048 (0.3760)
FLX	3.2509*** (3.3149)	-0.0719 (-0.1478)	-0.0044 (-0.0501)	0.5408 (1.0771)
Consume	-5.6004*** (-3.7326)	-0.3911 (-0.5226)	-0.1675 (-1.2422)	-1.2631 (-1.6394)
DIF	2.2366*** (5.2563)	0.0788 (0.3622)	0.1394*** (3.6441)	0.3511 (1.5714)
FA		0.1870*** (6.1619)		
AD				1.0479*** (2.9434)
Time fixed	Yes	Yes	Yes	Yes
Individual fixed	Yes	Yes	Yes	Yes
Observed value	300	300	300	300
R^2^	0.8565	0.8047	0.8878	0.7830

The results of the regression of digital inclusive finance on agricultural digitization are shown in Table 5 Model 3, where the coefficient of digital inclusive finance is positively significant. Add in the digital inclusive finance and agricultural digitization to make a return to the integration of rural industries, the results are shown in Table 5 Model 4, the coefficient of digital financial inclusion is insignificant and the coefficient of agricultural digitization is significant, indicating a full mediation effect, which verifies the previous hypothesis 3. Digital inclusive finance significantly enhances the agricultural digitization and reduces transaction costs and risks in agricultural markets [54,55], thus promoting the integration of rural industries.

### 4.5. Moderating effects analysis

To test the moderating effect of financial support, the interaction term between financial support for agriculture and digital inclusion finance was added to the explanatory variables. The regression results are shown in Table 6 Model 2, and compared with the baseline regression results in Table 6 Model 1, the core explanatory variables remained significantly positive and the interaction term was significantly negative, indicating that the moderating effect of financial support weakened the impact of digital inclusion finance on the integration of rural industries, which verified the previous hypothesis 4. The possible reasons for this are the relatively short period of financial support, the fact that the financial support mechanism is not yet perfect, and the inefficient use of financial funds, resulting in the failure of digital inclusive finance to play an effective role in the integration of rural industries.

**Table 6:** Regression analysis of the moderating effect of entrepreneurial education.

Variables	Model 1 RI	Model 2 RI
Constant	0.9345 (2.9901)	1.7759 (1.0295)
Electricity	0.2698*** (0.1005)	0.1934*** (3.6481)
UR	0.0212* (0.0117)	0.0186*** (4.0871)
FLX	0.5362 (0.5095)	0.8872** (2.2932)
Consume	-1.4386* (0.7795)	-1.3732*** (-2.6294)
DIF	0.4971* (0.2211)	0.5805** (2.4582)
LnGOV×DIF		-0.1964*** (-3.8285)
Time fixed	Yes	Yes
Observations	300	300
R^2^	0.5739	0.3469

### 4.6. Spatial variation analysis

The regressions were conducted separately for the eastern, central and western regions and the results are shown in Table 7. The regressions all passed the F-test, with R^2^ of 0.7071 for the eastern region, 0.8151 for the central region and 0.8565 for the western region. The fit of the regression is better in the western region, but the coefficient of digital inclusive finance in the western region is significantly negative, probably due to the underdeveloped digital infrastructure in the western rural areas, which has not brought into play the digital dividend, but rather the deepening of the digital divide, the poor infrastructure conditions for the integration of rural industries and the low financial literacy of farmers, resulting in a negative effect of digital inclusion on the integration of rural industries. The coefficients of digital inclusive finance in East and Central China are significantly positive, and digital inclusive finance has better promoted the development of local rural industrial integration. This is due to the better digital and financial infrastructure in East and Central China, the deeper integration of agriculture and secondary and tertiary industries, the higher financial demand generated by industrial integration, the extensive application of digital technology, the wide coverage of digital inclusive finance, and the sustained efforts to support the integration of rural industries [56,57]. The contribution of digital inclusive finance to the integration of rural industries shows regional heterogeneity, testing the previous hypothesis 5.

**Table 7:** Regression analysis of the eastern, central and western regions in China.

Variables	Model 1 East	Model 2 Central	Model 3 West
DIF	1.3083** (2.0310)	0.3433** (2.1092)	-1.4818*** (-3.5425)
Electricity	0.1598 (0.9944)	3.2838 (0.858317)	-2.6921 (-0.7444)
UR	0.0435* (1.8882)	0.0285* (1.6851)	0.0790*** (3.0016)
FLX	-0.5866 (-0.5751)	0.3729 (0.6879)	1.6760** (2.118820)
Consume	-0.1324 (-0.0687)	-0.0929 (-0.1321)	-1.4776 (-1.4423)
Constant	-7.6244 (-0.0921)	-4.7084* (-1.7820)	2.0959 (0.6014)
Time fixed	Yes	Yes	Yes
Individual fixed	Yes	Yes	Yes
Observed value	110	80	110
R^2^	0.7071	0.8151	0.8565

## 5. Conclusions and future prospects

### 5.1 Conclusion

This paper discusses how digital inclusive finance promotes the rural integration of rural industries, sorts out its role paths and influences intermediaries, explores whether financial support plays a mediating role, and further explores whether there is a significant difference in the impact of digital inclusive finance on the integration of rural industries in subsidized areas. The following conclusions are drawn: (1) The development of rural industrial integration is on a growing trend, with Jiangsu, Hainan and Shanghai provinces developing rural industrial integration more rapidly, and Guangxi, Jiangxi, Heilongjiang and Jilin provinces developing rural industrial integration more slowly. (2) The empirical results show that digital inclusive finance has a significant promoting effect on the integration of rural industries, with insignificant effects on the coverage breadth and using depth, a significant effect on the digitization degree, and a significant effect on raising per capital electricity consumption and urbanization rate can promote the development of industrial integration, the pulling effect of consumption on the integration of rural industries is limited, and the effect of per capital fixed asset investment on the integration of rural industries is not significant. (3) Digital inclusive finance influences the integration of rural industries by enhancing financial accessibility and agricultural digitization, and financial accessibility and agricultural digitization play a fully mediating role. (4) Financial support plays a negative moderating role in the relationship between the two. (5) The impact of digital inclusive finance on the integration of rural industries shows regional differences, with the eastern and central regions playing a significant role in promoting it and the western region showing a negative impact.

### 5.2. Implications

#### 5.2.1. Theoretical implications

The possible theoretical contributions of this paper are: analyzing the impact paths of digital inclusive finance on the integration of rural industries from the perspectives of financial accessibility and agricultural digitization, enriching the theoretical framework for the study of the relationship between the two, and providing theoretical support for digital inclusive finance to precisely promote integration of rural industries. The moderating role of financial support in the impact of digital inclusive finance on the integration of rural industries is explored, and financial support plays a negative moderating role in the relationship between the two, providing theoretical guidance to better utilize the advantages of digital inclusive finance and promote the integration of rural industries.

#### 5.2.2. Practical implications

Improve the integration of rural industries, achieve balanced development among regions, and force the development of digital inclusive finance with the new demand generated by industrial integration. Continue to give full play to digital advantages, enhance the degree of digitization of digital inclusive finance, promote digital inclusive finance products, and highlight the inclusive nature of digital inclusive finance. Strengthen the construction of power facilities in rural areas, improve the urbanization rate, stimulate consumption, adjust the structure of fixed assets, and promote the extension of industrial chain. Improve farmers’ credit awareness, optimize the construction of rural credit system, and improve the pertinence and effectiveness of financial popularization. Strengthen the construction of digital hardware facilities and network system in rural areas, increase the density of 5G base stations, and build a "digital village". Give play to the synergistic effect of finance and finance, promote the integrated development of rural industries as a whole, improve financial transparency and efficiency in the use of funds, and constantly adjust and optimize the structure and direction of financial capital investment. To implement the differentiated development strategy, the eastern region should play a role of radiation and assistance, and support the promotion of digital inclusive finance in the surrounding areas and the integration of rural industries. The central and western regions should increase basic financial service facilities such as bank outlets and POS machines, promote the integration of digital inclusive finance with local characteristic industries, and provide financial support for industrial development in remote areas.

### 5.3. Deficiencies and future prospects

The digital inclusive finance variables were selected by directly using the Peking University Digital Inclusive Finance Index, with data from the Ant Group system. The index portrays the overall situation of the development of digital inclusive finance, while this paper aims to explore the impact of digital inclusive finance on the integration of rural industries. In the future, we can draw on the Digital Inclusive Finance Index to screen relevant indicators and construct a rural digital inclusive finance index to provide more consistent data for the study of financial support for the development of agricultural industries. In addition, this paper only focuses on the advantages of digital inclusive finance, but the rapid development of digital inclusive finance as a new financial industry has also brought new risks, and it is still worthwhile to ponder on the regulatory issues and consumer rights protection, and whether it will bring new risks to the already fragile agriculture, which is worth further exploration in the future.

## Supporting information

S1 Dataset (ZIP)
